# Regulation of CTLA-4 and PD-L1 Expression in Relapsing-Remitting Multiple Sclerosis Patients after Treatment with Fingolimod, IFNβ-1α, Glatiramer Acetate, and Dimethyl Fumarate Drugs

**DOI:** 10.3390/jpm11080721

**Published:** 2021-07-27

**Authors:** Afshin Derakhshani, Zahra Asadzadeh, Hossein Safarpour, Patrizia Leone, Mahdi Abdoli Shadbad, Ali Heydari, Behzad Baradaran, Vito Racanelli

**Affiliations:** 1 Immunology Research Center, Tabriz University of Medical Sciences, Tabriz 516615731, Iran; a.derakhshani@oncologico.bari.it (A.D.); Zahraasadzadeh2834@gmail.com (Z.A.); abdoli.med99@gmail.com (M.A.S.); 2 IRCCS Istituto Tumori “Giovanni Paolo II” of Bari, 70124 Bari, Italy; 3 Cellular and Molecular Research Center, Birjand University of Medical Sciences, Birjand 9717853577, Iran; safarpour701@yahoo.com; 4 Department of Biomedical Sciences and Human Oncology, University of Bari “Aldo Moro”, 70124 Bari, Italy; patrizia.leone@uniba.it; 5 Student Research Committee, Tabriz University of Medical Sciences, Tabriz 516615731, Iran; 6 Department of Applied Mathematics, University of California, Merced, CA 95343, USA; aliheydari@ucdavis.edu; 7 Health Sciences Research Institute, University of California, Merced, CA 95343, USA; 8 Department of Immunology, Faculty of Medicine, Tabriz University of Medical Sciences, Tabriz 516615731, Iran

**Keywords:** CTLA-4, PD-L1, single-cell RNA-seq, PBMC, MS

## Abstract

Multiple sclerosis (MS) is a chronic demyelinating disease of the central nervous system (CNS) that is characterized by inflammation which typically results in significant impairment in most patients. Immune checkpoints act as co-stimulatory and co-inhibitory molecules and play a fundamental role in keeping the equilibrium of the immune system. Cytotoxic T-lymphocyte antigen-4 (CTLA-4) and Programmed death-ligand 1 (PD-L1), as inhibitory immune checkpoints, participate in terminating the development of numerous autoimmune diseases, including MS. We assessed the CTLA-4 and PD-L1 gene expression in the different cell types of peripheral blood mononuclear cells of MS patients using single-cell RNA-seq data. Additionally, this study outlines how CTLA-4 and PD-L1 expression was altered in the PBMC samples of relapsing-remitting multiple sclerosis (RRMS) patients compared to the healthy group. Finally, it investigates the impact of various MS-related treatments in the CTLA-4 and PD-L1 expression to restrain autoreactive T cells and stop the development of MS autoimmunity.

## 1. Introduction

Multiple sclerosis (MS) is the most prevalent non-traumatic disabling disease that causes demyelination of the central nervous system (CNS). A relapsing-remitting type of multiple sclerosis (RRMS) is the most pervasive type of disease. The etiology of MS is unknown, but biology, environmental conditions, and immune systems are all described as risk factors [1]. The prevalence of this neurodegenerative disease is due to an autoreactivity background composed of activated lymphocytes, macrophages, and microglia, which enter the CNS and lead to inflammation that can result in demyelination. Previous research showed that the immune system has a pivotal role in the pathogenesis of MS [2,3]. The immune system contains a potent set of effector mechanisms that, in addition to protecting the body against invasive pathogens, can also cause damage to the body itself. To avoid such tissue damage and restore inactivity following an inflammatory response, precise immune regulation is required. In the periphery, immune cell reactions are controlled by an equilibrium between stimulatory and inhibitory signals, attuning effector cells to their environment. In the case of T cells, these signals are delivered through numerous regulatory molecules, named immune checkpoints [4]. Among the diverse checkpoint therapies, Cytotoxic T-lymphocyte-associated protein 4 (CTLA-4) and Programmed death (PD-1)/Programmed death-ligand 1 (PD-L1) may be the most significant immune checkpoints for preventing autoactivation [5]. Substantial advancements in laboratory approaches and bioinformatics pipelines have allowed researchers to deconvolute highly complex immune cell populations in healthy and diseased states. For example, single-cell RNA sequencing (scRNA-seq) could be used to identify diverse and unusual cell populations, ascertain regulatory interactions between genes, and map the developmental trajectories of distinct cell lineages [6].

CTLA-4 from CD28 family receptors, by expressing on T cells, can regulate activation of T lymphocytes. CTLA-4 is primarily expressed by regulatory T cells (Tregs) and has a vital role in self-antigen tolerance and inhibition of autoimmunity [7,8]. CTLA-4 negatively regulates T cell activation in numerous mechanisms. CTLA-4 and CD28 compete for binding to B7-1 and B7-2 on APCs. Thus, the binding of the B7 to CTLA-4 of T-cells causes the inhibition of the activity of T-cells. It has been shown that the lack of CTLA-4 can trigger autoimmune conditions in murine models, so CTLA-4 is considered a critical factor in regulating both central and peripheral tolerance [9]. Therefore, deficiency of CTLA-4 can be correlated with the development of autoimmune diseases such as multiple sclerosis [10].

PD-1 is another immune checkpoint, and their ligands including, PD-L1 and PD-L2, have a prominent role in the inhibition of T cell signaling and facilitating immune homeostasis and tolerance. Growing evidence reveals that loss of PD-1/PD-L can be involved in autoimmunity [11]. A recent study showed that immune checkpoints such as PD-1, CTLA-4, and TIM-3 have a significant reduction in MS patients compared to the healthy group [12].

There are notable changes in multiple sclerosis therapy due to the introduction of molecular effects of medications. Many drugs have been approved for MS treatment, including IFNβ-1α, glatiramer acetate, and fingolimod [13], but the results of these drugs on immune checkpoints have not been clarified yet. Here, we evaluated the effect of CTLA-4 and PD-L1 expression in regulating T-cell tolerance and autoimmunity in RR-MS patients to uncover the possible relationship of these immune checkpoints in the pathogenesis of RR-MS. In this study, we first applied a systems biology approach for analyzing single-cell RNA-seq data of MS patients and healthy controls that characterized the presence of CTLA-4 and PD-L1 in the different cell populations. Next, we analyzed the expression value of CTLA-4 and PD-L1 in 40 peripheral blood mononuclear cell (PBMC) samples from RR-MS patients receiving various drugs, including Fingolimod, Interferon-beta 1-alpha (IFNβ-1α), Glatiramer acetate (GA), and Dimethyl fumarate (DMF), and compared their expression results with 5 naïve PBMC samples as well as 16 healthy donor PBMC. 

## 2. Materials and Methods

### 2.1. In Silico Study

#### 2.1.1. Data Acquisition, Quality Control, and Dimensionality Reduction

We obtained the data used for this study from research by Schafflick et al. [14]. Researchers in the initial study used single-cell transcriptomics of blood and CSF cells from patients with MS and controls. The raw data from their research on single-cell RNA-seq have been deposited in the Gene Expression Omnibus (GSE138266) [15]. The Scanpy toolkit [16] was leveraged for data analysis. First, quality control was performed to filter low-quality cells. To do this, we only kept cells that had (I) more than 500 genes, (II) less than 17,500 counts, and (III) less than 20% of reads mapped to mitochondrial genes.

On the other hand, genes that are expressed by a minimum of 30 cells were kept. Cell count was normalized with a scaling factor of 10,000, whereas gene expression was scaled to unit variance and a mean value of 0. To allow unsupervised clustering and organization of cell types, dimensionality reduction was performed with the top 4000 most highly variable genes with true biological variability (FDR < 0.01) from technical noise using a quantitative statistical method [17] for principal component analysis (PCA). PCA on the combined set of samples for each sample after selection of highly variable genes. Once embedded in this PCA space, we constructed a nearest neighbor graph, identifying the *k* = 15 nearest neighbors for each cell. We derived uniform manifold approximation (UMAP) embeddings presented for visualization from this most relative neighbor graph using a minimum distance of 0.5 and a spread of 1.0 [18]. The Louvain method was then used to detect similar cell populations. The Louvain’s resolution parameter was set to 0.5. Genes were then ranked using *scanpy.api.tl.rank_genes_groups* (Benjamini–Hochberg, *t*-test overestimated variance with adjusted *p*-value). Cell types were manually and iteratively assigned based on overlaps of literature-curated and statistically ranked genes. 

#### 2.1.2. Clustering Cells and Cell-Type Identification

For this work, we used Louvain community detection [19] to the nearest neighbor graph constructed in PCA space to define a cluster partition. Marker genes are typically identified through their differential expression (DE) between clusters, as the more strongly DE genes are more likely to have produced separate clustering of cells. To measure the differences in expression profiles, numerous different statistical tests can be used.

### 2.2. Experimental Study

#### 2.2.1. Patient and Control Groups

In total, 45 patients (32 females and 13 males) aged 20 to 59 years old who had been diagnosed with RRMS according to McDonald’s criteria [20] were recruited in this study. This study was approved by the Tabriz University of Medical Sciences Ethics Committee (IR.TBZMED.REC.1399.074), and all participants were made aware of the information they received before participating. None of the patients had prior therapy for RRMS. Patients were excluded if they had primary or secondary MS progression, having other chronic CNS degenerative disorder, or inflammatory, or autoimmune disease. Sixteen healthy individuals were included as a control group (8 females and 8 males). Both control and treatment groups were chosen from the same ethnicity and geographical zone.

#### 2.2.2. Blood Sampling and Isolation of Mononuclear Cells from Peripheral Blood

PBMCs were separated using Ficoll-Hypaque gradient centrifugation according to the manufacture’s instruction (Lymphodex, Inno-Train, Germany), as mentioned in the previous paper [13].

#### 2.2.3. RNA Isolation, cDNA Synthesis, and Quantitative Real-Time PCR

Total RNA was extracted from PBMCs using TRIzol reagent (GeneALL Biotechnology CO., LTD, Seoul, Korea), and cDNA was synthesized using cDNA synthesis kit (Biofact, Korea). Real-time PCR was performed using 2X Master Mix with high ROX (Biofact, Korea) for the quantification of CTLA-4 and PD-L1 with the following primers: CTLA-4, forward: CATGATGGGGAATGAGTTGACC, reverse: TCAGTCCTTGGATAGTGAGGTTC; PD-L1, forward: TGCCGACTACAAGCGAATTACTG, reverse: CTGCTTGTCCAGATGACTTCGG, appropriate amounts of template cDNA, and primer sets under the following condition: initial denaturation 13 min, 95 °C, 45 cycles of denaturation; 13 s, 95 °C; annealing, 30 s, 59 °C; elongation 20 s, 72 °C. All gene expression was normalized with Glyceraldehyde-3-phosphate dehydrogenase (GAPDH) (F:5′-AAGGTGAAGGTCGGAGTCAAC-3′, R: 5′-GGGGTCATTGATGGCAACAA-3′) as an internal control. The relative level of CTLA-4 and PD-L1 mRNA expression was determined with the 2^−∆*C*T^ method.

#### 2.2.4. Statistical Analysis

Python 3.7 was used for the single cell sequencing analysis. Statistical analysis was performed using GraphPad Prism 8.0.0 software (San Diego, CA, USA). We chose a significance threshold of 0.05 (probabilities under 0.05 were considered statistically significant).

## 3. Results

### 3.1. Single-Cell Transcriptome Analysis

#### 3.1.1. Differential Cell-Type Proportion Analysis

**Figure 1:**
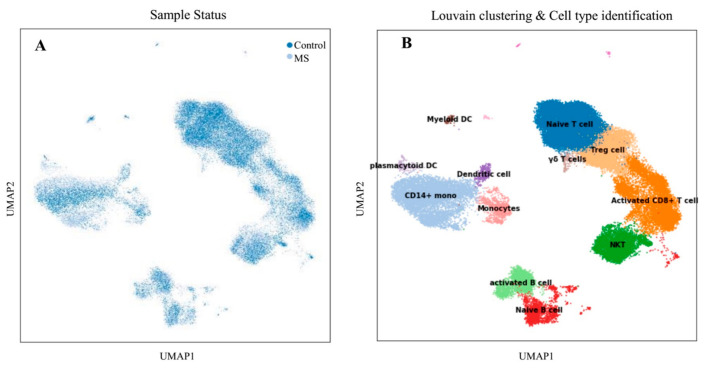
Transcriptomic comparison of MS versus control PBMCs. (**A**) UMAP projection of cells with the normal situation (*n* = 17,138) colored in dark blue and cells from MS samples (*n* = 25,831) were visualized in light blue. (**B**) Louvain clustering and cell annotation was employed to identify 12 specific cell populations.

Transcriptome profiling was conducted on various sub-groups of PBMCs samples to identify molecular signatures associated with each cell type. Schafflick et al. [14] recently reported the molecular signature of MS pathogenicity in CSF and PBMCs samples using single-cell RNA sequencing technologies. Since most of the cells in this dataset are PBMS cells, this dataset provides a valuable resource for understanding the expression situation of our hub-genes that selected from previous sections. We used the Scanpy package version 1.7 [15] to re-analyzed the scRNA-seq data. After data pre-processing, a total of 40,515 cells remained. Louvain clustering and cell annotation were used to identify major cell populations. We measured the distribution of the total number of cells in each cluster between those in the MS and the normal population. As shown in Figure 1, 12 different cell types were identified based on the specific markers between control and MS patients. 

#### 3.1.2. Visualization of CTLA-4 and PD-L1 Genes in a Single Cell Resolution

**Figure 2:**
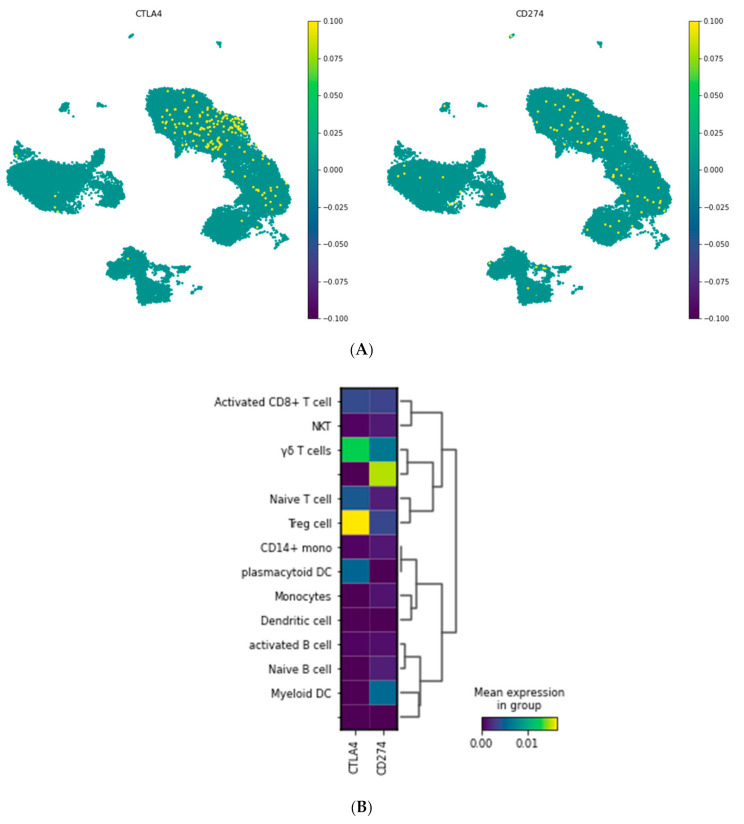
CTLA-4 and PD-L1 (CD274) expression in the different cell populations of PBMCS. (**A**) Embedding of the graph in two dimensions using UMAP; (**B**) MatrixPlot shows the CTLA-4 and PD-L1 expression in the diverse cell populations. As is shown, CTLA-4 is mainly expressed in Treg cells and gamma delta T cells, while PD-L1 is not expressed in a specific cell type.

To visualize the expression of CTLA-4 and PD-L1 in various cell types and to better understand their role, the expression values of these genes were visualized on subgroups of PBMCs using UMAP (Figure 2). CTLA-4 and PD-L1 were expressed mainly on the naïve T cells, regulatory T cells, and activated CD8^+^ T cells.

### 3.2. Experimental Study

#### 3.2.1. Clinical Characterization of the Study Population

A total of 40 RRMS patients who received IFNβ-1α (*n* = 10), fingolimod (*n* = 10), DMF (*n* = 10), or GA (*n* = 10) as routine MS medicine therapy for at least 3 months were compared to five RR-naïve MS patients and 24 healthy controls. We illustrated the baseline characteristics of the study population in Table 1.

#### 3.2.2. Comparison of CTLA-4 Expression between MS Patients and Controls

**Figure 3:**
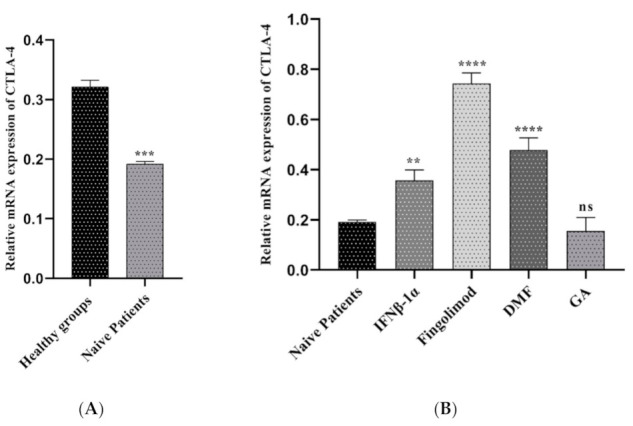
The relative expression of CTLA-4 between MS patients and controls; (**A**) healthy groups and naïve RRMS patients, (**B**) naïve RRMS patients and Fingolimod-, DMF-, GA-, and IFNβ-1α-treated patients (** *p* ≤ 0.01. *** *p* ≤ 0.001. **** *P* ≤ 0.0001. ns: not significant).

Expression levels of CTLA-4 were determined in the sample of different under-treatment MS patients and compared with naïve patients and healthy groups. As shown in Figure 3A, CTLA-4 expression was significantly high in controls compared with naïve patients (0.32 vs. 0.19, *p*-value = 0.0004). The expression value of CTLA-4 in the pre-treated groups was different based on the drug which they took. The relative expression of CTLA-4 in patients treated with Fingolimod was far higher than naïve patients (0.74 vs. 0.19, *p* < 0.0001). Furthermore, the group which received DMF and IFNβ-1α showed the increased expression value compared to the naïve ones (0.47 vs. 0.19, *p* < 0.0001; 0.35 vs. 0.19, *p* < 0.0001 respectively). In contrast, the relative expression of CTLA-4 in the treated groups with GA were lower than naïve patients; additionally, it was not statically significant (0.15 vs. 0.19, *p* = 0.6809) (Figure 3B).

#### 3.2.3. The PD-L1 Expression in MS Patients and Controls

**Figure 4:**
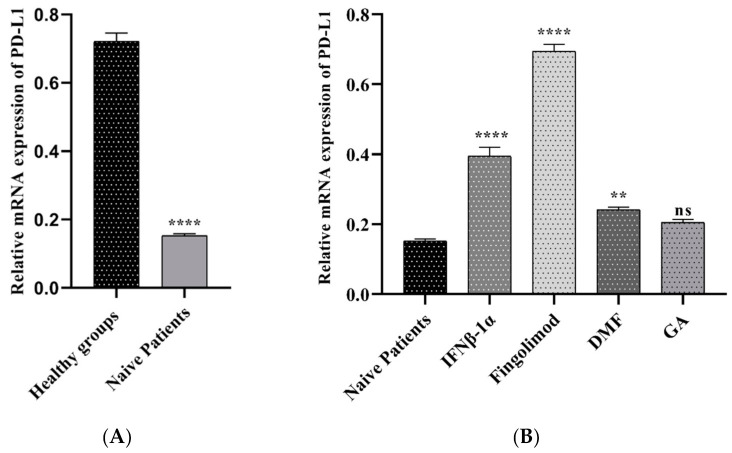
The relative expressions of PD-L1 mRNAs in PBMCs between MS patients and controls; (**A**) healthy groups and naïve RRMS patients, (**B**) naïve RRMS patients and Fingolimod-, DMF-, GA-, and IFNβ-1α-treated patients (** *p* ≤ 0.01. **** *p* ≤ 0.0001. ns: not significant).

To identify the role of PD-L1 gene expression in the pathogenesis of multiple sclerosis, we used the real-time PCR method. As shown in Figure 4A, PD-L1 was increased significantly in the healthy subjects compared to naïve patients at mRNA level (0.72 vs. 0.15, *p* < 0.0001). This study found a significant upregulation of PD-L1 gene expression in the PBMCs of patients treated with Fingolimod than naïve patients (0.69 vs. 015, *p* < 0.0001). Pre-treated patients with GA and IFNβ-1α showed a similar pattern to the fingolimod group, but the increased level was different (0.39 vs. 0.15, *p* < 0.0001; 0.24 vs. 0.15, *p* < 0.0001 respectively). The upregulation was detected in the patients who used GA, although it was not statically significant (Figure 4B). 

## 4. Discussion

MS is a common chronic autoimmune disease that affects CNS by demyelination. Although the exact cause of MS is unknown, many studies point to genetics and environmental factors that might contribute to the disease progression. It is reported that several immune cells have a pivotal role in the development of MS, and in particular, T cells as the most identified cell type [21,22].

Newly, scRNA-seq techniques allow us to annotate non-classified cells solely based on the mRNA expression patterns of each cell. The massive amount of biological data acquired from scRNA-seq leads us to organize cells into particular groups, analyze their heterogeneity, predict the functions of sc populations based on the gene expression profiles, and find out the cell proliferation or development pathways. Recently, researchers, by using the scRNA-seq technique, suggested a strategy for specific targeting to delay or prevent the progressive phase of MS [23]. The immune system developed inhibitory receptors to restrict unnecessary T cell-mediated inflammatory reactions, termed immune checkpoints. The inhibitory checkpoint points to T cells’ various co-receptors that negatively regulate T cells and significantly preserve peripheral self-tolerance. CTLA4 and PD-L1 are the most potent examples of these checkpoint molecules and are critical in maintaining immunologic homeostasis [24].

CTLA-4 has a significant effect in regulating immune hemostasis and inhibition of autoimmunity [24]. Lack of CTLA-4 leads to an autoimmune condition in mice described by polyclonal T cell proliferation that confirms an essential impact for CTLA-4 regulating T cell responses. Genetical analysis has proved an association between single-nucleotide polymorphism (SNP) of the CTLA-4 gene and susceptibility to MS. The first (SNP), which related to MS susceptibility, is placed at position +49 (G > A) in exon 1 of the CTLA-4 gene [25]. Additionally, it is suggested that the dysregulated CTLA-4 expression in MS patients and T-cell responses could result, at least in part, from variations at the genetic level. Karabon et al. indicated that the gene polymorphisms of CTLA-4 are related to the level of CTLA-4 expression in MS patients and susceptibility to disease [26]. Moreover, Viglietta et al. demonstrated that preventing co-stimulatory molecule interactions using CTLA4Ig appears protected in MS [25]. The immunologic effects suggest that regulating the inflammatory response related to MS might be a promising strategy [27].

PDL-1 is the ligand of PD-1, belongs to the B7 family, and is expressed on T lymphocytes, B lymphocytes, and antigen-presenting cells. The primary function of the PD-1/PD-L1 pathway is to restrain the T cell activity in peripheral tissues during the inflammatory reaction to infection and stop autoimmunity [28,29]. It is indicated that PD-1 and PD-L1 expression in PBMCs from MS patients was considerably lower than the healthy controls. PD-1 and PD-L1 downregulation might suggest that over-stimulation of immune cells in MS happens via signaling dysfunction of these molecules [30].

We used an online sc RNA-seq dataset to characterize CTLA-4 and PD-L1 expression in different PBMC cell types. Our single-cell sequencing results showed that CTLA-4 and PD-L1 have a substantial expression in the naïve T cells, Tregs, and activated CD8^+^ T cells. Regulatory T cells are crucial players in maintaining immune tolerance due to their ability to regulate autoreactive T cells. Therefore, these cells show a central role in stopping surplus autoreactive immune responses [31]. Furthermore, we considered CTLA-4 and PD-L1 expression in PBMC samples of healthy groups and RRMS patients who did not receive any treatment (naïve patients). As presented in Figure 3A and Figure 4A, the results reported that CTLA-4 and PD-L1 expression in naïve patients is lower than in healthy individuals. We also measured the expression of CTLA-4 and PD-L1 genes in PBMC samples of RR-MS patients who received various treatments of Fingolimod, IFNβ-1α, GA, and DMF and compared them with samples of naïve patients (Figure 3B and Figure 4B).

Fingolimod is an approved treatment for RRMS. It functions as a functional antagonist of the sphingosine-1-phosphate (S1P) receptor, which sequesters lymphocytes in lymph nodes and prevents them from entering the CNS [32]. A recent study assessed the in vivo impacts of Fingolimod on T cells and Tregs of RR-MS patients that received Fingolimod for 12 months. It is stated that Fingolimod has a direct effect on the phenotype of T cells and Tregs. Additionally, it decreases pro-inflammatory cytokines such as TNFα and the number of peripheral T cells. Effector CD4^+^ T cells exposed a reduced expression of Interleukin 17 (IL-17) and IFNγ; however, a growing production of TGFβ and IL-10 and increased the expression of factors related to exhausted T cells PD-1 and Tim-3 [33].

Moreover, Baer et al. identified the effect of Fingolimod on human T cell receptor signaling pathways [34]. They proposed that besides the S1PR-related regulation of T cell response, Fingolimod leads to abnormal activation of NFAT1, AP1, and NF-κB, stimulating epigenetic alterations in human T cells, resulting in the suppression of T cell activation [34]. To evaluate the effect of Fingolimod on CTLA-4 and PD-L1 expression, we used the real-time PCR method. Interestingly, we found that treatment with Fingolimod significantly increases the expression of both CTLA-4 and PD-L1 in the PBMC of RRMS patients compared to the naïve MS patients (Figure 3B and Figure 4B).

IFNβ-1α is another treatment that we considered in this study. It is a cytokine in the interferon family used to treat MS, and it is reported that interferons lead to about an 18–38% reduction in the rate of MS relapses. It is demonstrated that IFN-β triggers the proliferation of CD4^+^CD25^+^Foxp3^+^ Tregs by upregulation of GITRL on dendritic cells in the MS treatment [35]. To evaluate the IFNβ-1α effect on CTLA-4 and PD-L1 expression, we used PBMC samples of IFNβ-1α-treated MS patients and confirmed that both CTLA-4 and PD-L1 expression increases in these groups in comparison with naïve patients. Additionally, the results indicated that the IFNβ-1α effect in the PD-L1 expression is more significant than the CTLA-4 expression.

DMF was introduced as a novel orally accessible disease-altering factor for the controlling of RR-MS. A study directed by Gross et al. revealed that DMF has substantial effects on memory T cells accompanied by a change in T helper cell populations in MS patients, leading to an alteration toward anti-inflammatory reactions [36]. Researchers found that DMF decreases absolute lymphocyte counts, but does not affect all subsets uniformly. CD8^+^ T cells were the most intensely affected. Still, a decrease also occurred in the CD4^+^ T cells, mainly within the pro-inflammatory T-helper Th1 and Th17 groups, generating a bias toward more anti-inflammatory Th2 and regulatory groups [37]. In this work, we evaluated DMF’s impact on the expression of both immune checkpoint molecules, CTLA-4 and PD-L1. The results of real-time PCR indicate that DMF-treated samples have a high expression of PD-L1 and CTLA-4 compared to naïve patients.

Furthermore, we examined the influence of GA on CTLA-4 and PD-L1 expression in these patients. GA is a polypeptide-dependent therapy accepted for the RR-MS treatment [38]. GA therapy is supposed to support Th_2_-polarized GA-reactive CD4⁺ T-cells development, reducing adjacent inflammation within the CNS. Current data signify that CD4⁺ CD25⁺ FoxP3⁺ Tregs deficiency in MS is restored by GA treatment [38]. However, our study shows that GA-treated samples have no significant effect on CTLA-4 and PD-L1 expression.

Finally, our findings suggest that Fingolimod can be the most effective treatment for MS patients. The mechanism of action of Fingolimod can be associated with its ability to induction of these two immune checkpoint expressions. In GA, the induction of CTLA-4 and PD-L1 expression is not an acceptable mechanism in treating RR-MS patients. 

The current study has some strengths and weaknesses. In terms of strengths, we demonstrated CTLA-4 and PD-L1 expression in various immune cells using the novel approaches of bioinformatics and validated the result by Real-time PCR. For the first time, we showed the effects of the approved drugs on the CTLA-4 and PD-L1 expression in PBMCs. Our study had some drawbacks as well. We only evaluated CTLA-4 and PD-L1 expression at the mRNA level and could not work on the protein level.

## 5. Conclusions

Based on our experiments, we find that various treatments, especially Fingolimod, induce the expression of inhibitory checkpoints, CTLA-4 and PD-L1, and increased expression or function of these molecules can result in the decreased responses of autoreactive T cells and lead to the inhibition of autoimmune diseases, including MS. More studies and techniques are needed to reveal the exact mechanism of the abovementioned immune checkpoints in the pathogenesis of MS.

## Figures and Tables

**Table 1:** Characteristics of patients and controls.

Groups	Fingolimod (*n* = 10)	IFNβ-1α (*n* = 10)	DMF (*n* = 10)	GA (*n* = 10)	Naïve Patients (*n* = 5)	Healthy Control (*n* = 6)
Age (Mean ± SD)	34.3 ± 6.1	35.1 ± 10.3	28 ± 6	33.7 ± 7.2	34 ± 5	30.1 ± 7.4
Female *n* (%)	7 (70%)	7 (70%)	7 (70%)	7 (70%)	4 (80%)	8 (50%)

Abbreviations: Interferon-beta 1-alpha (IFNβ-1α), Glatiramer acetate (GA), Dimethyl fumarate (DMF). SD: standard deviation.

## Data Availability

The dataset which used in this study was previously published and can be accessed via the Gene Expression Omnibus (GEO) website (https://www.ncbi.nlm.nih.gov/geo/ (accessed on 1 July 2021)) under the GSE accession number referred to above in the Methods section.
